# Rheumatoid Arthritis with Deficiency Pattern in Traditional Chinese Medicine Shows Correlation with Cold and Hot Patterns in Gene Expression Profiles

**DOI:** 10.1155/2013/248650

**Published:** 2013-09-23

**Authors:** Minzhi Wang, Gao Chen, Cheng Lu, Cheng Xiao, Li Li, Xuyan Niu, Xiaojuan He, Miao Jiang, Aiping Lu

**Affiliations:** ^1^Institute of Basic Research in Clinical Medicine, China Academy of Traditional Chinese Medicine, Beijing 100700, China; ^2^School of Life Sciences, Hubei University, Wuhan 430062, China; ^3^Sino-Japan Friendship Hospital, Beijing 100029, China; ^4^School of Chinese Medicine, Hong Kong Baptist University, Kowloon, Hong Kong; ^5^E-Institute of Shanghai Municipal Education Commission, Shanghai TCM University, Shanghai 201203, China

## Abstract

In our precious study, the correlation between cold and hot patterns in traditional Chinese medicine (TCM) and gene expression profiles in rheumatoid arthritis (RA) has been explored. Based on TCM theory, deficiency pattern is another key pattern diagnosis among RA patients, which leads to a specific treatment principle in clinical management. Therefore, a further analysis was performed aiming at exploring the characteristic gene expression profile of deficiency pattern and its correlation with cold and hot patterns in RA patients by bioinformatics analysis approach based on gene expression profiles data detected with microarray technology. The TCM deficiency pattern-related genes network comprises 7 significantly, highly connected regions which are mainly involved in protein transcription processes, protein ubiquitination, toll-like receptor activated NF-**κ**B regulated gene transcription and apoptosis, RNA clipping, NF-**κ**B signal, nucleotide metabolism-related apoptosis, and immune response processes. Toll-like receptor activated NF-**κ**B regulated gene transcription and apoptosis pathways are potential specific pathways related to TCM deficiency patterns in RA patients; TCM deficiency pattern is probably related to immune response. Network analysis can be used as a powerful tool for detecting the characteristic mechanism related to specific TCM pattern and the correlations between different patterns.

## 1. Introduction

Rheumatoid arthritis (RA) is a common autoimmune disease with a prevalence of over 1% of the population worldwide [1]. This inflammatory disease is characterized by chronic inflammation and progressive impair in joints and immunity disorder [2, 3]. Inflammatory cell infiltration in synovial membrane plays a crucial role in the pathogenesis of RA, including CD4+ T cells, which are activated in joint sites [4–7]. Activated CD4+ T cells can promote IL-17 producing and enhance immune activity, and other cytokines like IL-2, IL-4, and IL-10 are also secreted by CD4+ T cells, and these will either upregulate or downregulate immune reaction in RA patients [8–10]. Circulating CD4+CD161+ T cells are now regarded to be a potential biomarker of RA disease activity [11]. Thus, it is believed that studies on this subset of T cells can promote better understandings of joint immune response and inflammation in RA.

Patients with RA vary considerably in terms of their clinical manifestations and outcome [12]. Some of the complementary and alternative medical systems, such as traditional Chinese medicine (TCM), take a different approach in diagnosing and treating RA. In TCM, patients with RA are further stratified according to their symptoms, a pattern (also called Zheng or syndrome) diagnosis will be determined for a subgroup of the patients, and then specific therapy will be prescribed based on these pattern classifications [13]. In RA patients, cold, hot, and deficiency patterns were regarded as 3 major pattern classifications in clinical practice [14]; in our previous study, the correlation between cold and hot patterns in TCM and gene expression profiles in RA has been explored [15]. MAPK signaling, Wnt signaling, and insulin signaling pathways were revealed to be related to TCM hot pattern; purine metabolism was related to both TCM hot and cold patterns; alanine, aspartate, and tyrosine metabolisms were related to TCM cold pattern, and histidine metabolism and lysine degradation were related to TCM hot pattern [15]. Interestingly, other studies also detected the functions of general cold/hot pattern-related genes [16, 17], and in some specific diseases, such as gastritis [18], further found the imbalanced network biomarkers for traditional Chinese medicine syndrome in gastritis patients [19]. These results indicated that different pattern could be regarded as a consequence of biological networks comprising hundreds of thousands of gene expressions changed in various affected tissues and immune effector cells; thus, the functional gene network analysis might be a useful tool in exploring the biological fundamentals of pattern classification in RA.

As another key pattern diagnosis among RA patients, deficiency pattern can lead to a specific treatment principle of “tonifying the deficiency” in clinical management. Therefore, the elucidation of the underlying mechanism of deficiency pattern in RA in the context of gene expression profile should not only open out mechanism of classical TCM theory on pattern classification but also enriche current research on complex diseases. 

Yet the study on deficiency pattern should be different from that on cold/hot pattern, and for that the hot and cold patterns are typical, these two types of patterns can be easy to be classified in each patient in clinical practice. The deficiency pattern summarizes another set of clinical manifestations, and in most cases it is accompanied with hot or cold pattern (resulting in cold-deficiency pattern, or hot-deficiency pattern). Thus in order to achieve better understanding on deficiency pattern and the comparison with cold/hot pattern, in this study we concentrated on the symptom sets by factor analysis which contribute to pattern classification and then we try to explore the significantly related genes to these symptom sets. This analysis method has been successfully used in our previous study on cold and hot patterns [15]. 

Therefore, on the basis of previous study, further analysis aiming at exploring the characteristic gene expression profile of deficiency pattern and its correlation with cold and hot patterns in RA patients is underscored. In the present study, a genome-wide expression technology and systems biology approach were combined to reveal the characteristic gene expression profile of deficiency pattern and its correlations with cold and hot patterns in RA patients.

## 2. Materials and Methods

### 2.1. Patients

A total of 33 female RA patients from the China-Japan Friendship Hospital and 12 healthy female volunteers from the China Academy of Chinese Medical Science in Beijing, aged 18 to 70 years old, participated in the study. RA patients were eligible to participate if they had met the American College of Rheumatology (ACR) criteria for RA for at least one year, with functional classes of I, II, or III [20]. Patients with cold/hot/deficiency pattern, or combination pattern (hot deficiency or cold deficiency), were recorded with whole clinical manifestations according to TCM theory using a questionnaire, a tongue examination, and pulse diagnosis by 3 appointed TCM practitioners [14, 21]. Patients were included in the study only if the 3 practitioners reported consistent results. Healthy females without any diagnosed diseases were included as controls.

Patients who had continuously received nonsteroidal anti-inflammatory corticosteroid drugs for more than 6 months or who had received these medicines within one month were excluded from the study. Patients with severe cardiovascular, lung, liver, kidney, mental, or blood system diseases and women who were pregnant, breastfeeding, or planning pregnancy in the next 8 months were excluded from the study. A complete joint function and biochemical function evaluation was available for all participants in the study. The study was granted by the local Ethical Committee for Clinical Research, and each patient signed informed consent before enrollment. 

### 2.2. Sample Preparation

For the microarray, 8 mL of venous blood was collected in anticoagulation tubes from each of the 45 participants before breakfast. CD4+ T cells were extracted and purified using the RosetteSep Human CD4+ T Cell Enrichment Cocktail (StemCell Technologies, Inc., Vancouver, Canada) [22]. Total RNA was isolated from the CD4+ T cells using the Trizol extraction method (Invitrogen, Carlsbad, Canada), as described by the manufacturer. mRNA was amplified linearly using the MessageAmp aRNA Kit (Ambion, Inc., Austin, USA), in accordance with the instructions of the manufacturer. cRNA was purified using an RNeasy Mini Kit (QIAGEN, Hilden, Germany) based on a standard procedure.

### 2.3. Microarray Assay

Total RNA was extracted using TRIZol reagent from CD4+ T cells according to the manufacturer's instruction. Probes were verified for amplification yield and incorporation efficiency by measuring the DNA concentration at 280 nm, Cy3 incorporation at 550 nm, and Cy5 incorporation at 650 nm. For each color, 10 pmol incorporated dye was fragmented and resuspended in 500 *μ*L hybridization solution. Samples were hybridized to dual-color human Whole Genome Microarray (University of British Columbia, Canada) that contained four arrays of probes representing around 23,232 well-characterized transcripts. The arrays were hybridized in microarray hybridization chambers overnight at 42°C. After washing, the slides were scanned with GenePix 4000B scanner. 

All nonflagged array elements for which the fluorescent intensity in each channel was 1.5 times greater than the local background were considered well measured. The ratio values were log-transformed (base 2) and stored in a table (rows, individual cDNA clones; columns, single mRNA species). cDNA spots that fulfilled the intensity criteria on at least 80% of the microarrays were analyzed. Data for the remaining genes were centered by subtracting (in log space) the median observed value to remove any effect of the amount of mRNA in the common reference pool. 

### 2.4. Statistics and Functional Analysis

#### 2.4.1. TCM Pattern Classification and Correlation Analysis

Since clinical manifestation is the key information for TCM pattern classification, and in most cases the deficiency pattern is accompanied with hot or cold pattern, in this study, 58 clinical manifestations were listed for recording before blood collection based on TCM theory. Then factor analysis was applied for clustering the clinical manifestations into 3 different sets based on the TCM pattern diagnosis. The 3 factors contributed significantly to the cold, hot, and deficiency pattern diagnoses, respectively; furthermore, clinicians proved that the symptoms in each factor were the most common ones for diagnosing the relative pattern in real TCM clinical practice. Therefore, the factors were regarded as the representatives of the 3 patterns and employed in further correlation analysis. The TCM patterns-related genes were selected with correlation analysis (coefficient > 0.5 or <−0.5, and *P* < 0.05 as significant). All data were analyzed on an SAS9.1.3 statistical package (Order no. 195557), and a cluster analysis was performed using Cluster 3.0 and Tree View software.

#### 2.4.2. Microarray Statistics

The data were normalized to correct for technical variations among individual microarray hybridizations using the two-step procedure described in detail by Jarvis and colleagues. The signal intensity of each expressed gene was globally normalized (LOWESS) using the R statistics program [23]. Any ratio between two groups (RA and healthy control) of more or less than 1 : 1.5 was taken as the differential gene expression criteria. Statistical significance was tested using the Student's *t*-test (*P* < 0.05). Changes greater than 1.5-fold (cold or hot or deficiency pattern to control group) were recorded as upregulations, and those less than 1.5-fold (cold or hot or deficiency pattern to control group) were recorded as downregulations. Other fold changes for gene expression were recorded as normal expression. Changes in gene expression (1.5-fold change) were required in more than 50% of the patients. A chi-squared test was used for these comparisons (*P* < 0.05) and to identify similar and different genes in the deficiency pattern and cold/hot pattern groups of differentially expressed genes. Gene assemblages related to the 3 symptom sets (as 3 patterns) were obtained using correlation analysis. 

#### 2.4.3. Protein-Protein Interaction Analysis and Network Illustration

The information on human protein-protein interactions was obtained from databases, including Biomolecular Interaction Network Database (BIND), The General Repository for Interaction Datasets (BioGRID), Database of Interacting Proteins (DIP), Human Protein Reference Database (HPRD), Database system and analysis tools for protein interaction data (IntAct), and Molecular Interactions Database (MINT), and was complemented with curated relationships parsed from the literature using Agilent Literature Search [24]. These datasets are mostly based on experimental evidence. We did not include data that were deemed to be of lower quality. The protein-protein interaction network was visualized using cytoscape [25].

#### 2.4.4. Highly Connected Clusters of the Integrated Network

The database and the literature data mining networks were integrated, and then IPCA was used to analyze the characteristics of the network. The IPCA algorithm can detect densely connected regions in the interactome network [26]. Interactomes with a score greater than 2.0 and at least four nodes were taken as significant predictions in this study.

#### 2.4.5. Gene Ontology Analysis

To identify the function of each cluster generated by IPCA individually, GO clustering analysis was performed with the proteins described in all subnetworks. For this purpose, the latest version of Biological Network Gene Ontology (BiNGO) tool [27] was used to statistically evaluate groups of proteins with respect to the existing annotations of the Gene Ontology Consortium. The degree of functional enrichment for a given cluster was quantitatively assessed (*P* value) by hypergeometric distribution, implemented in BiNGO tool. The 10 GO biological categories with the smallest *P* values were selected as significant.

## 3. Results

### 3.1. The Identification of Deficiency Pattern

In the 33 enrolled patients (12 with cold pattern and 21 with hot pattern), 18 were diagnosed with deficiency pattern and 15 with non-deficiency pattern. Among the 18 patients with deficiency pattern, 8 were diagnosed with cold-deficiency pattern and 10 with hot-deficiency pattern. No typical deficiency pattern was individually diagnosed. The basic clinical information of the enrolled patents was listed in Table 1. The clinical manifestations of RA patients were clustered into three sets with factor analysis, which were corresponding to the cold, hot, and deficiency patterns in TCM, respectively. Deformity, inhibited bending and stretching in limbs, pain occurring or worsening at night, pain occurring or worsening during moodiness, and numbness were categorized in TCM deficiency pattern (as listed in Table 2). The symptom sets for cold and hot patterns are the same as those reported in our previous cold and hot patterns in RA study (data not shown) [14].

### 3.2. The TCM Deficiency Pattern and Its Functional Gene Networks

The TCM RA deficiency pattern-related genes were selected using correlation analysis. All related genes were listed in Table 3.

**Figure 1:**
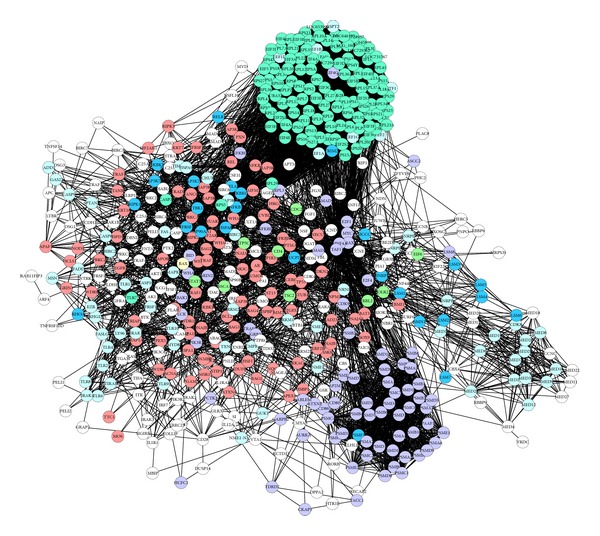
Protein-protein interaction (PPI) network for deficiency pattern-related genes. Cycles represent nodes. All edges represent interactions between the nodes. The green nodes represent cold pattern-related molecules; plum stands for hot pattern-related molecules; light blue stands for deficiency pattern; yellow stands for both cold and hot patterns; bluish green stands for both cold and deficiency patterns; deep blue stands for both hot and deficiency patterns; dark blue stands for cold, hot, and deficiency patterns.

**Figure 2:**
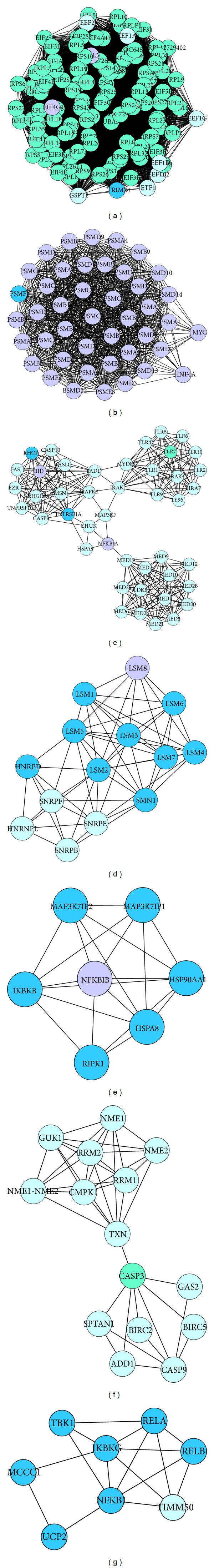
The subnetworks made up of highly connected regions and functions of the nodes in TCM deficiency pattern-related genes. Cycles represent nodes. All edges represent interactions between the nodes. Clusters with score >2 were considered to be significant (it represents the log of the probability that the network was found by chance).

To further refine the biological functions of these genes, protein and protein interaction analysis was applied based on the genes listed in Table 3, and the molecular networks for those genes were built up (Figure 1). In the network, the nodes represent proteins and the edges represent interactions between the proteins. More importantly, during the process of analysis, cold and hot patterns related differential genes (data not shown) were marked in the deficiency pattern network if those cold or hot pattern-related genes were included in the network in order to clarify the potential relations among deficiency pattern and cold/hot pattern in RA. Seven significantly, highly connected regions were proposed by IPCA, and these subnetworks of highly connected regions were visualized by cytoscape (Figure 2).

The subnetworks of highly connected regions and functions of the nodes are mainly involved in protein transcription processes, protein ubiquitination, toll-like receptor activated nuclear factor *κ*B (NF-*κ*B) regulated gene transcription and apoptosis, RNA clipping, NF-*κ*B signal, nucleotide metabolism-related apoptosis, and immune response processes. Particularly the articular manifestations in deficiency pattern were related to toll-like receptor activated NF-*κ*B regulated gene transcription and apoptosis (Figure 2(c)) and nucleotide metabolism-related apoptosis (Figure 2(f)).

### 3.3. TCM Deficiency Pattern-Related Central Subnetwork Analysis and Its Correlations with Cold and Hot Patterns

**Figure 3:**
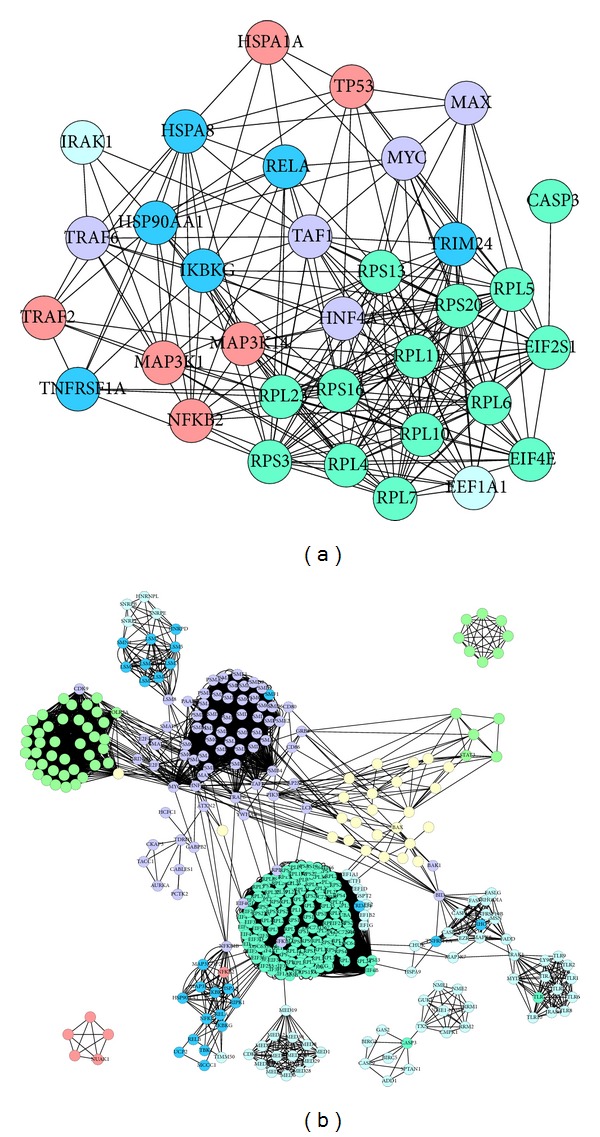
(a) Central PPI subnetwork related to deficiency pattern in RA. (b) PPI network from combined modules of cold, hot, and deficiency patterns-related genes. Cycles represent nodes. All edges represent interactions between the nodes. The green nodes represent cold pattern-related molecules; plum stands for hot pattern-related molecules; light blue stands for deficiency pattern; yellow stands for both cold and hot patterns; bluish green stands for both cold and deficiency patterns; deep blue stands for both hot and deficiency patterns; dark blue stands for cold, hot, and deficiency patterns.

The deficiency pattern-related central subnetwork was analyzed by IPCA, and the functions of genes involved in the central subnetwork were presented in Table 4. The central subnetwork and functions of the nodes are mainly involved in NF-*κ*B and macromolecule metabolic processes. The central subnetwork was visualized by cytoscape (Figure 3(a)). Most nodes in this network are dark blue, bluish green, plum and deep blue, which indicate that the major biological mechanism of TCM deficiency pattern is closely correlated with cold and hot patterns.

Then the network modules of deficiency, cold, and hot patterns are combined (Figure 3(b)), and through network analysis, the correlations among deficiency, cold, and hot patterns are revealed. There are 3 green-predominant modules which represent cold pattern-related mechanism, and functions of these modules are mainly involved in ubiquitylation, RNA clipping, and JAK-STAT cascade signaling; 1 plum-predominant module represents hot pattern related mechanism with function of insulin signaling; 3 light blue-predominant modules are deficiency pattern related, with functions of toll-like receptor activated NF-*κ*B regulated gene transcription and apoptosis, and nucleotide metabolism related apoptosis; 1 yellow-predominant module is related to both cold and hot patterns with function of JAK-STAT signaling-related apoptosis; 1 bluish green-predominant module is related to both cold and deficiency patterns with function of protein translation process; 2 deep blue-predominant modules denote the mechanism relevant to both hot and deficiency pattern, with functions of NF-*κ*B signaling and mRNA clipping; 1 dark blue-predominant module is related to cold, hot, and deficiency patterns, with the functions of ubiquitylation and apoptosis. 

## 4. Discussion

The major findings in this study include revealing the characteristics of TCM deficiency pattern and the correlations between deficiency pattern and cold/hot pattern in gene expression profile. To date, this is the first study to explore the distinct and common features among three basic patterns in RA patients at a genome-wide level. 

From the view of both biomedicine and TCM, the clinical manifestations in RA patients are diverse and complex, which pose challenges on diagnostic study. For example, it has been pointed out that, besides some common articular symptoms, bone destruction or erosion-related symptoms can start early or later from disease onset, which can result in loss of physical function [28]. Given the tight correlation between physical disability and loss of social and economic opportunities in patients with RA [29], treatment options which are capable of profoundly interfering with joint damage would appear to be of greater value under the framework of biomedical theory [30]. 

In TCM clinical practice, the symptoms of RA can be classified into different groups, and based on this classification, pattern diagnosis can be determined and guides the therapy selection [13]. The cold, hot, and deficiency patterns are key patterns in RA patients, and they can be identified individually or concomitantly, such as deficiency-cold, deficiency-hot, or intermingled cold and hot patterns. The pattern classification can be regarded as a further subgrouping of patients, with the aim of a more precise individualized treatment. Thus, with the ultimate aim to preserve functional status and joint architecture in RA patients, better understanding and characterization of the underlying mechanism of TCM pattern classification in RA patients have been an important goal in RA research in the present years. 

Given the complexity of pattern differentiation in clinical practice, another distinctive point in this study is that we selected symptom factors, instead of the typical patients with some specific patterns, as representatives of TCM patterns in the correlation analysis. The significant predominance of this method lies in that we can avoid the confusion resulting from multiple-patterns diagnosis occurring in one patient and the disagreement of different clinicians. Aiming at the major symptom sets which contribute to a pattern would better concentrate on the pattern diagnosis itself and the comparison with other concomitant patterns. This method has been successfully used in our previous study on correlation between cold and hot patterns in gene expression profile [14]. With cold feeling in joints and pain relieved with warming which are important for TCM cold pattern differentiation and hot feeling and pain relieved with cooling which are important for TCM hot pattern differentiation [14], this classification has been verified with factor analysis [31]. For TCM deficiency pattern identification, deformity and inhibited bending and stretching in limbs are key manifestations, and generally these symptoms clinically occur in the later stage of disease or later than other common articular symptoms such as pain with cold or hot feeling and swelling. Thus, in most cases, deficiency pattern is believed as result of prolonged disease or delayed treatment. Of course in some aged patients, deficiency pattern can be identified from the disease onset because of the weak general state of health. What is more, the deficiency pattern is often diagnosed and complicated with cold or hot pattern in clinical practice. These diagnostic principles are in line with the corresponding understandings of RA in biomedicine, also accordant with our findings in this study. 

The articular manifestations in deficiency pattern of RA patients were found to be related to protein transcription processes, protein ubiquitination, toll-like receptor activated NF-*κ*B regulated gene transcription and apoptosis, RNA clipping, NF-*κ*B signal, nucleotide metabolism-related apoptosis, and immune response processes. In particular, toll-like receptor activated NF-*κ*B regulated gene transcription and apoptosis and nucleotide metabolism-related apoptosis were specifically responsible for deficiency pattern identification. 

NF-*κ*B is a set of multifunctional transcriptional factors that regulate expression of genes involved in numerous normal cellular activities [32–34]. They were activated in many inflammatory and neoplastic conditions in which their expression may be stimulated by proinflammatory cytokines [35, 36]. NF-*κ*B regulated the expression of cytokines and mediated autocrine, self-amplifying cycles of cytokine release, and NF-*κ*B activation leading to maintenance of inflammatory reactions beyond the initial stimulus in RA [36, 37]. Since discovery of the requirement of NF-*κ*B for basal and cytokine-induced osteoclast formation in the mid-1990s, many studies have been focused on the role of NF-*κ*B in bone [38–40]. NF-*κ*B plays predominant roles both in skeletal development, endochondral ossification, osteoclast, and osteoblast functions [39, 41] and in synovial cells of RA [42, 43]. In the synovial cells of patients with RA, activation of the NF-*κ*B pathway resulted in the transactivation of a multitude of responsive genes that contribute to the inflammatory phenotype, including TNF-*α* from macrophages, matrix metalloproteinase from synovial fibroblasts, and chemokine that recruit immune cells to the inflamed pannus. In short, inhibition of NF-*κ*B pathway is believed to be a potential therapeutic target in RA [44]. 

Toll-like receptor is one of the key functions of innate immune system, which can recognize both exogenous pathogen-associated molecular patterns (PAMPs) and endogenous molecules created upon tissue injury, sterile inflammation, and degeneration [45]. Endogenous toll-like receptor ligands were called as damage-associated molecular patters (DAMPs), including endogenous molecules released by activated and necrotic cells and extracellular matrix molecules [46]. The molecule family had many members. Synovial tissues from RA joints expressed toll-like receptor 2 predominantly at attachment sites and invaded cartilage and bone, mostly in synovial fibroblasts, but not in macrophages, in which expression was enhanced not only by IL-1*β* and TNF-*α* but also by LPS [47]. In synovia of patients with early stage RA, increased expression of toll-like receptors 3 and 4 was demonstrated as well as that of toll-like receptors 2, 3, and 4 in long-lasting RA synovitis [48]. This finding might provide a potential and partially explanation on why deficiency pattern can be identified in both later and early stages of RA. Toll-like receptor activation triggers intracellular signaling pathways that lead to the induction of inflammatory cytokines, type I IFNs, and upregulation of costimulatory molecules leading to the activation of the adaptive immune response [49]. Toll-like receptor activation can promote angiogenesis in various inflammatory settings in response to both exogenous and endogenous ligands [50]. Crucially, further investigation showed that toll-like receptors were important modulators of mesenchymal stem cells; which played great role in bone formation and bone remodeling, and important regulators in angiogenesis [51, 52]. These findings could partially explain why this pathway is tightly related to TCM deficiency pattern identification, which is based on the onset of joint deformity and inhibited bending and stretching in limbs symptoms. 

**Figure 4:**
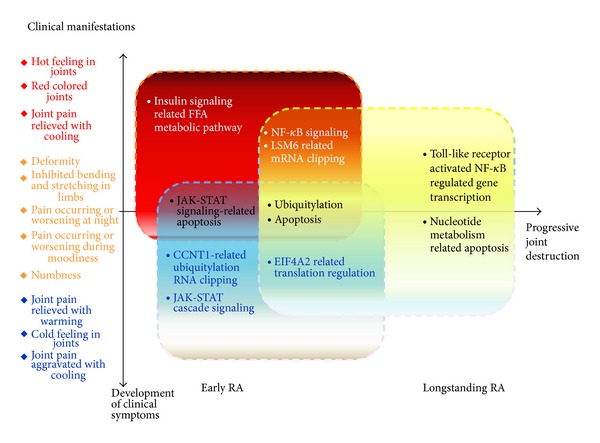
Correlations and characteristics among TCM deficiency, cold, and hot patterns in RA concerning pathways and clinical manifestations. Blue square denotes cold pattern, red square represents hot pattern, and yellow square indicates deficiency pattern.

Since the deficiency pattern is often diagnosed and complicated with cold or hot pattern in clinical practice of TCM, it is of tantamount importance to understand the correlations between deficiency pattern and cold/hot pattern. In our study, the network analysis provides some clues about answering this question. The clinical manifestations and relevant pathways of TCM cold, hot, and deficiency patterns in RA are presented in Figure 4. The results further support our previous findings that different patterns were related to different pathways; on the other hand, it is also indicated that some pathways were shared in different patterns, which might be the underlying mechanism of shared symptoms in different patterns or intermingled patterns. Our data also imply that the identification of the underlying mechanism of and correlations among the TCM patterns might contribute to better understanding of pathogenesis of RA.

However, several potential limitations in our study should not be neglected. Firstly, the typical RA patients with single deficiency pattern are hard to be identified, and in most cases deficiency pattern occurs accompanied with other patterns such as hot or cold pattern. Thus the symptom sets of these patterns acquired by factor analysis were adopted in the gene expression analysis, which might result in some indeterminateness and variance, although this method has been successfully applied in previous study on cold and hot patterns in RA patients. Secondly, our study population is relatively small, and some bias might exist; for example, all enrolled patients are female, despite the exploration of distinct pathways of each TCM pattern and their correlations. Further study of larger sample size is needed in the future. In addition, there exist other TCM patterns in RA patients besides cold, hot, and deficiency patterns, and the neglecting of these patterns might have potential impact on our results.

## 5. Conclusions

Toll-like receptor activated NF-*κ*B regulated gene transcription and apoptosis and nucleotide metabolism-related apoptosis pathways are potential specific pathways related to TCM deficiency pattern in RA patients, and TCM deficiency pattern is probably related to immune response. Network analysis can be used as a powerful tool for detecting the distinct and common characteristics among different patterns in RA patients at molecular level. These findings are also able to contribute to the pathogenesis study in RA in light of systems biology.

## Figures and Tables

**Table 1:** Basic information of enrolled patients.

Index	Value range	Mean ± SD
Age (years)	25–55	42.8 ± 9.9
Duration of RA (month)	1–240	57.4 ± 56.9
X-ray score	0–2	0.18 ± 0.47
Rheumatoid factor	0–673	135.6 ± 177.0
Erythrocyte sedimentation rate (mm/h)	4–140	43.9 ± 35.6
C-reactive protein (mg/L)	0–185	13.0 ± 34.1

**Table 2:** The factor related to deficiency pattern obtained in factor analysis after oblique PROMAX rotation*.

Clinical symptoms	Factor loading
Deformity	0.81
Inhibited bending and stretching in limbs	0.69
Pin occurring or worsening at night	0.63
Pain occurring or worsening during moodiness	0.58
Numbness	0.40

*The data in the table are factor loadings obtained after oblique PROMAX rotation. The factor loading represents the correlation power of the symptom with the factor. A loading value of more than 0.20 suggests that there is correlation of the articular manifestation with the factor.

**Table 3:** TCM deficiency pattern related genes in RA*.

Name	ID	Coefficient value	*P* value
Hypothetical gene CG018	NM_052818	0.61	0.0045
Homo sapiens cDNA FLJ11557 fis, clone HEMBA1003083	AK021619	0.59	0.0011
Hypothetical protein FLJ10769	NM_018210	0.58	0.0057
Homo sapiens cDNA: FLJ23020 fis, clone LNG00943	AK026673	0.55	0.0027
Cytochrome c-1	NM_001916	0.53	0.0116
Phosphoprotein associated with glycosphingolipid-enriched microdomains	NM_018440	0.53	0.002
Tumor necrosis factor receptor superfamily, member 10b	AF016266	0.53	0.012
Hematological and neurological expressed 1	NM_016185	0.53	0.0015
Homo sapiens cDNA FLJ13721 fis, clone PLACE2000450	AK023783	0.53	0.0044
HSPC041 protein	NM_016099	0.53	0.0016
KIAA0826 protein	AB020633	0.52	0.0046
Small fragment nuclease	NM_015523	0.52	0.0169
Protein phosphatase 2A 48 kDa regulatory subunit	NM_013239	0.51	0.0096
HSPC160 protein	NM_014182	0.51	0.0051
Mitochondrial ribosomal protein L11	NM_016050	−0.5	0.0046
Hypothetical protein MDS025	NM_021825	−0.51	0.0028
KIAA1270 protein	AB033096	−0.56	0.0104
DKFZP586D0824 protein	BC013934	−0.56	0.0008
Lin-7b protein; likely ortholog of mouse LIN-7B; mammalian LIN-7 protein 2	NM_022165	−0.64	0.0019

*The genes were selected with correlations analysis (coefficient value >0.5 or <−0.5, and *P* < 0.05).

**Table 4:** Description of central subnetwork biofunctions related to deficiency pattern in RA.

Description	*P* value
Translational elongation	9.4409*E* − 19
Cellular protein metabolic process	7.6789*E* − 13
Cellular macromolecule metabolic process	2.2814*E* − 11
Cellular macromolecule biosynthetic process	9.7307*E* − 11
Macromolecule biosynthetic process	1.3144*E* − 10
Macromolecule metabolic process	5.9427*E* − 10
Cellular metabolic process	1.0405*E* − 09
Gene expression	1.8878*E* − 09
Primary metabolic process	4.6891*E* − 09
Activation of NF-*κ*B-inducing kinase activity	1.4532*E* − 08
